# Utilization of Rosmarinic and Ascorbic Acids for Maturation Culture Media in Order to Increase Sow Oocyte Quality Prior to IVF

**DOI:** 10.3390/molecules26237215

**Published:** 2021-11-28

**Authors:** Oana-Maria Boldura, Simona Marc, Gabriel Otava, Ioan Hutu, Cornel Balta, Camelia Tulcan, Calin Mircu

**Affiliations:** 1 Faculty of Veterinary Medicine, Banat’s University of Agricultural Sciences and Veterinary Medicine “King Michael I” Timisoara, 300645 Timişoara, Romania; oanaboldura@usab-tm.ro (O.-M.B.); simona.marc@usab-tm.ro (S.M.); gabrielotava@usab-tm.ro (G.O.); ioanhutu@usab-tm.ro (I.H.); calinmircu@usab-tm.ro (C.M.); 2 BUASVM’s Research Institute for Biosecurity and Bioengineering, University of Agricultural Sciences and Veterinary Medicine ”King Michael I of Romania” from Timisoara, 300645 Timişoara, Romania; 3 Institute of Life Sciences, Vasile Goldis Western University of Arad, 310414 Arad, Romania; baltacornel@uvvg.ro

**Keywords:** antioxidants, oocytes, in vitro maturation, apoptosis, sow

## Abstract

The beneficial effect of antioxidant supplementation in maturation culture media of sow oocytes was evaluated by the expression quantification of apoptotic genes and the genes that ensure stability of germ cells during fertilization. The oocytes were cultivated for 44 h in conventional medium (C) or in medium supplemented with 105 µM rosmarinic acid (R) and 0.5 mM ascorbic acid (A) and classified into three quality classes by morphological observation from which the total RNA was isolated. The gene expression of Ptx3 and the apoptotic regulator p53, Bax and BCL-2 were evaluated by quantitative PCR technique. The decreased expression of the Bax gene in the A and R groups, compared to the control, indicates a protective role of antioxidants in the cells. Cell homeostasis was maintained, as reflected in the ratio of Bax/Bcl-2 in class I COCs (cumulus-oocyte complex) regardless of the experimental group, indicating minimum cellular stress. The expression of p53 genes was higher in all class III COC, but in A1 and R1 the expression was lower than in C1, and a similar Ptx-3 gene decreased significantly in groups A1, A2, A3 and R1 compared with control groups. Antioxidant supplementation showed beneficial effects on all morphological classes of pig COCs.

## 1. Introduction

Throughout recent years, swine-assisted reproductive techniques with in vitro fertilization have been used in research primarily for studies on mammalian embryogenesis, xenotransplantation, transgenesis, and genome editing. However, the methods used are not always standardized and must be further improved.

Embryo technologies involve the use of assisted reproduction, of cellular and molecular biology, and genomics. Their use in research aims to solve questions about endocrine control, molecular changes and metabolic pathways that regulate embryonic development, and in animal husbandry aims to increase the number of products with a superior genotype.

The worldwide demand for animal products is steadily increasing, and there is a need to use advanced technologies to increase both the quantity and the quality of production. In the last decade, in vitro fertilization (IVF) has developed from an experimental science into a technology that is extensively used in bovine species [1,2], but in swine the large scale implementation of IVF techniques is still poor due to polyspermy and low embryo development, even if there are studies with promising results [3,4].

Ensuring in vivo oocyte growth and maturation are essential steps for the oocyte to gain the ability to grow into an embryo and later a healthy newborn. In order to increase the quality and quantity of oocytes, the current procedures focus mainly on improving the homogeneity of the oocyte population in terms of growth at the beginning of a treatment or on the modification of hormone’s quantity added in the maturation media [5].

Cellular alterations through the action of reactive oxygen species are the most damaging to normal cellular development. Reactive oxygen species (ROS) are generated by numerous endogenous and exogenous sources and have a major impact on reproduction and fertilization [6]. ROS plays an important role in oocyte maturation, in ovarian steroidogenesis, in folliculogenesis, ovulation, luteolysis, implantation and blastocyst development. Oxidative metabolism is indispensable to produce energy in the ovarian follicle, which in turn generates ROS [6].

Although their presence is essential for physiological activities, too much ROS causes oxidative stress and affects mitochondria and cellular structures such as lipid membranes, nucleic acids and proteins. Preventing oxidative stress is essential to maintaining normal reproductive functions. The source of ROS during assisted reproduction techniques may be endogenous in gametes or may be exogenous by environmental factors (visible light, culture medium, pH, temperature, oxygen concentration, centrifugation, cryopreservation) [7].

The use of natural compounds from plants can improve and can also be an alternative source of reducing oxidative stress. The properties of plant extracts or various metabolites have been analyzed in the cultivation of various cell types, including follicles and granulosa cells. L-ascorbic acid, a hydrosoluble antioxidant and electron donor plays an important role in multiple biological processes to reduce free radicals and reactive oxygen species by serving as a powerful antioxidant (non-enzymatic function), and as an essential cofactor to modulate the family of ferrous ion and 2-oxoglutarate (Fe^2+^ and 2-OG)-dependent dioxygenases (enzymatic function) [8].

In the practice of IVF ascorbic acid has often been used in different concentrations as a supplement in in vitro culture systems for embryos but also for the maturation of domestic cat oocytes [9], buffalo and bovine oocytes [10,11], canine oocytes [12] or pig oocytes [8]. The better results in their studies were with 50 µg/mL ascorbic acid in bovine oocytes [11], 50 µM for buffalo oocytes [10], 250 µM for canine oocytes [12], or 250 µM for pig oocytes [8]. In sperm cryopreservation protocols, ascorbic acid has given really good results due to its antioxidant capacity, especially with a concentration of 3 mg/mL, or a concentration of 5 mM [13] for bull semen or a concentration of 600 µM in human semen during vitrification [14].

The epigenetic effect of ascorbic acid is well known based on studies proving its role as enhancer of Tet enzyme activity, enzymes that convert 5-methylcytosine to 5-hydroxymethycytosine and, thus activating the gene, also by promoting the demethylation of histone H3 lysine 9 (H3K9) by improving somatic cell reprogramming during iPSC [15,16]. Epigenomic remodeling is also induced by ascorbic acid in myeloid leukemia [17]. Transfer of ascorbic acid within the nucleus of porcine somatic cell nucleus is used to improve embryo development with results based on higher levels of reprogramming gene transcripts, such as Pou5fl, Sox, and Klf [18]. As a result of reduced oxygen tension, the embryos acquire improved development, evident by a decrease in reactive oxygen species (ROS) content and in DNA fragmentation. Considering that ROS species will affect maturing oocytes and ultimately trigger them to apoptosis, the expression of genes involved in the execution and regulation of apoptosis namely Bcl-2 for maturated oocytes was quantified, with a significant increase in its expression in swine COC cultivated in cysteine supplemented medium, which means that this gene can be used as a molecular marker to evaluate oocyte viability [19,20].

Another antioxidant used as adjuvant in IVF is rosmarinic acid. Rosmarinic acid (α-o-caffeoyl-3,4-dihydroxyphenyllactic acid) is a natural compound found in species belonging to the Lamiaceae and Boraginaceae families [21]. Due to its constituents, namely rosmanol, carnosol, carnosic acid, ursolic acid, rosmariquinone, caffeic acid, rosmaridiphenol, flavonoids, diterpenes, steroids and triterpenes, rosemary extract and its derivatives have a variety of biological activities that include antioxidant, antimutagen, antibacterial, antiviral, anti-inflammatory, antiangiogenic, antitumor neuroprotective, antiallergic, anti-photoaging, hepatoprotective effects [21,22,23,24]. Natural extracts such as rosemary extracts are used as an oxidation protector in polymer formulations of polymers [25]. Zaharesco and Blanco (2021) studying various compounds with antioxidant properties (rosemary, POSS, polyhedral oligomeric silsesquioxane, quercetin, capsaicin, oleanolic acid) on the oxidation delay of the ethylene-propylene-diene monomer showed that prevention of oxidative degradation is related to the susceptibility of antioxidant molecules to the inactivation of free radicals. In the food industry, the standardized extracts of dried rosemary leaves have been approved by the European Union (E392) and the United States as a natural antioxidant [26]. In meat and meat products, rosemary is used as a food preservative because it prevents oxidation of lipids and protein oxidation [26]. Also, if rosemary and blackcurrant extracts are used in a broiler diet (2.5 and 5 g/kg in basal diets for 35 days), they tend to decrease malondialdehyde (MDA) concentration in thigh muscles during cold storage (4 °C) even if oxidative stability of frozen chicken meat was not affected [27]. The radioprotective capacity of rosmarinic acid has been observed by decreased frequency of micronuclei and by increases in cell survival determined by the MTT assay; effects attributable to its ability to scavenge ROS [28]. Due to low content availability of rosmarinic acid from natural sources and to such a variety of biological activities, artificial pathways for its production in heterologous systems as micro-organisms are considered. Omics future studies should be used to understand the metabolite synthesis pathways of rosmarinic acid [29].

This antioxidant acts as a free radical scavenger in biological systems, and could provide benefits for maturing oocytes, although in the literature there is only one article proving that rosmarinic acid, at a concentration of 5 µM, improved the development competence of porcine oocytes during the IVM period [30]. In semen cryopreservation research rosmarinic acid is also used with good results in maintaining the DNA integrity of ram [28] and boar sperm during preservation protocols [31,32], with best results when used in 105 µM concentration or on human sperm cryopreservation by freeze-drying technique [33]. For the complete evaluation of the health status of maturated oocytes, measurement of apoptotic gene expression has proven to be an extremely useful tool. In this molecular process several pro- and anti-apoptotic proteins are optimal candidates in offering a complete evaluation. The Bcl2 gene family consists of about 20 homologous pro- and anti-apoptotic regulators of programmed cell death. The Bcl2 protein prevents apoptosis and maintains cell survival by influencing mitochondrial cytochrome C release, being an anti-apoptotic factor. Bax (a pro-apoptotic factor), can suppress the ability of Bcl2 to block apoptosis [34]. The intrinsic mechanism of cellular apoptosis, including the Bcl2 protein family, is also present in ovarian granulosa cells, and the Bcl2/Bax ratio is an important aspect in the apoptotic process of granulosa cells [35]. The p53 protein plays a fundamental role in the repair processes of cellular DNA, being activated when the compensatory mechanisms go beyond the apoptotic process. The p53 gene and the p53 protein family is responsible for the genomic integrity of the germline [36]. In oocyte maturation, the p53 gene has been found to activate in response to stressors and therefore is an ideal marker for quantifying the viability of oocytes and embryos [37]. Ptx3 is a protein that ensures the formation, organization and stability of the extracellular cumulus matrix, through the mediation of the production and architecture of hyaluronan polysaccharides, secreted by cumulus cells [38].

Based on the role of these genes (Bax, Bcl2, p53 and Ptx3) in ensuring the quality of female gametes, the purpose of this research was to evaluate the antioxidant effects of ascorbic acid and rosmarinic acid supplementation in maturation culture medium on sow oocytes through quantification of the expression of the Bax, Bcl2, p53 and Ptx3 genes.

## 2. Materials and Methods

All chemicals were purchased from Sigma-Aldrich (Saint Louis, MO63103, USA), unless otherwise indicated.

### 2.1. Collection of Sow Cumulus-Oocyte Complexes

The ovaries (n = 60) were obtained from a local slaughterhouse and were transported within two hours in a 0.9% NaCl solution, ensuring a temperature of 33–35 °C. The oocytes were harvested by puncturing and aspirating the foliculles content with 18G needles. The follicular liquid is aspired to the 5 mL syringe and then introduced into 50 mL sterile tubes containing Dulbeco-PBS (D8662). On day of use, Dulbeco-PBS was supplemented with 1 mg/mL glucose, 0.036 mg/mL sodium piruvate, 0.02 mg/mL penicillin, 0.04 mg/mL streptomycin, 0.011 mg/mL heparin and 0.3 mg/mL BSA (bovine serum albumin). After 10 min the sediment was removed using sterile Pasteur pipettes and transferred to Petri dishes containing PBS, for the two cycles of washing.

The total number of COCs obtained, 393, were classified according to their morphological aspects using a stereomicroscope (Stemi 2000-C, ZEISS, Jena, Germany) with hot plate (33.4 °C) as follows; 1st class, COCs with compact and unexpanded cumulus, with full or at least 5 layers of cumulus cells, clearly seen cytoplasm, dense and homogeneous; 2nd class, COCs with compact cumulus, thick, 2–4 layers of cumulus cells, covering all of zona pellucida, dense cytoplasm, with uniform granulation; and 3rd class, oocytes partially denuded of cumulus cells, or with 1–2 complete layers of cumulus cells and/or with irregular shrunken cytoplasm.

### 2.2. In Vitro Maturation of Sow Cumulus-Oocyte Complexes

The maturation culture medium was prepared in our laboratory after Parrish et al. (1986) protocol with minor modifications: TCM 199 HEPES modification medium, (M2520) with 10% estrus cow serum (ECS) and 0.88UI/mL FSH (F8174); group C (control), in experimental groups we added 105 µM rosmarinic acid (536954)); group R and 0.5 mM ascorbic acid; group A, concentrations chosen based on the best results from literature obtained by other molecular evaluation [32,34,39]. The number of COCs submitted to in vitro maturation according to the morphological classification was 81 COCs class 1, 98 COCs class 2 and 214 COCs class 3. The 20–25 COC pools were matured in 400 µL media in 4-well dishes (Nunc, ThermoFisher Scientific, Roskilde, Denmarkcovered with mineral oil at 38.5 °C in 5% CO_2_ humidified air atmosphere for 44 h. After 44 h of culture, all COC were examined for maturation, signs such as expansion and mucification of the cumulus cells were observed. COC’s were matured according to their class (C1, C2, C3—control groups, R1, R2, R3—rosmarinic acid groups and A1, A2, A3—ascorbic acid groups).

### 2.3. Molecular Analysis of In Vitro Maturated Sow Cumulus-Oocyte Complexes

Before proceeding with RNA isolation, the cell samples were washed with PBS buffer and sediment by centrifugation at 3000× *g* for 5 min. For each sample approximately 1.5 × 10^3^ cells were harvested and submitted to the isolation protocol. After in vitro maturation, all COCs according to their morphological class and their experimental groups were subjected to RNA extraction, the median number of COCs class 1/sample was 27, COCs class 2/sample was 32.66 and COCs class 3/sample was 71.33. Total ARN was isolated from sedimented cells using the commercial kit SV Total RNA Isolation System (Promega, Madison, WI, USA) according to the manufacturer’s protocol.

From isolated RNA, the cDNA was synthesized using high-capacity cDNA reverse transcription (Thermo Fisher Scientific, Vilnius, Lituania) following the manufacturer’s indications and oligo dT(8) primer, also provided with the kit. The obtained cDNA was used as a template in the qPCR reaction using the GoTaq qPCR Master Mix Kit (Promega, Madison, WI, USA) following the instructions provided with the kit.

The RT-qPCR reactions were run with with Stratagene Mx3000P (Agilent Technologies, Santa Clara, CA, USA) real-time PCR equipment. The primers used in this study and their annealing tempertures were selected from the reference literature: Bax—sense 5′ GCT GAC GGC AAC TTCA ACT G 3′ and antisense 5′CCG ATC TCG AAG GAA GTC CA3′; Bcl2—sense 5′GGG ACG TCA GGT CAC TGA AT 3′ and antisense 5′ GAA ACC CCT AGT GCC ATC AA 3′; Reference gene β-actin—sense 5′ GTG ATC TCC TTC TGC ATC CTG TC 3′ and antisense 5′ CTC GAT ATG AAG TGC GAC G 3′ [40]; P53—sense 5′ GCA ATG GAT GAG GCG CAG TC 3′ and antisense 5′ TGG CAC TCT GGA GGC GTC AT 3′ [37]; and Ptx 3—sense 5′ TCA GGA ACA ATG TGA CCC GT 3′ and antisense 5′ TCA GCT ATC AGT CCA CTG GC 3′ [37]. The primers sequences were synthesized by Eurogentec (Amsterdam, Belgium).

Each sample was analyzed in triplicate. For normalization of gene expression in terms of the number of copies β-Actin gene was used. For each pair of primers, a sample without DNA template considered as negative control was analyzed. For all the samples, the number of cycle thresholds (Ct) was determined and the relative quantification of gene expression was achieved by using the Δ (ΔCt) method [41]. According to this method, R (the relative ratio between the control and stressed variant) is calculated with the following formula: R = 2^−ΔΔ^Ct.

### 2.4. Statistical Analysis

Data were entered into an Excel database (Microsoft Corp., Redmond, WA, USA). Descriptive statistics were generated to describe the frequency and the ratio between groups or classes. Statistical analysis was performed using IBM SPSS Statistics for Windows, Version 20.0. (IBM Corp., Armonk, NY, USA). Nonparametric tests, for independent samples were used for group comparisons.

## 3. Results

After morphological evaluation of porcine oocytes, we observed an increase in COC maturation in experimental groups compared with the control group. Therefore, after in vitro maturation of porcine oocytes in the medium supplemented with ascorbic acid (group A), 96.29% of class 1 COCs (A1) were matured after 44 h, 91.17% of class 2 (A2) and 12.98% of class 3 (A3), in the R group 96.42% of class 1 COCs (R1) were matured after 44 h, 63.33% of class 2 (R2) and 31.57% of class 3 (R3) compared with groups without antioxidant (group C) where we observed that 88.46% of class 1 COCs (C1), 20.58% of class COC 2 (C2) and 6.94% of class 3 COCs (C3) were matured.

**Figure A1:**
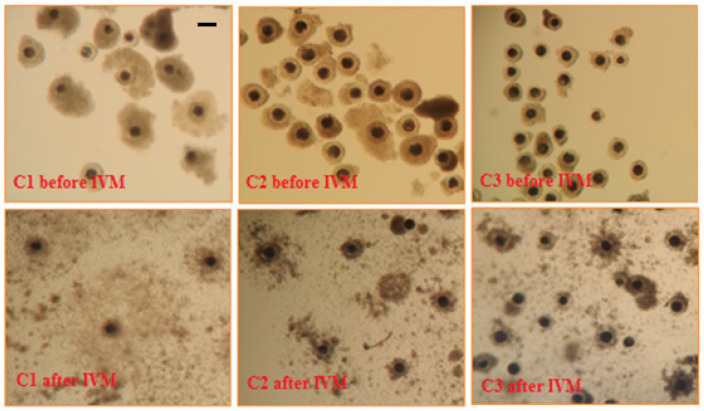
Aspects of porcine oocytes from control group (C) classified according to their morphological aspects (class 1, 2 or 3) before and after IVM. Scale bar represents 100 µM. The scale bar is applicable to all panels (3.2×).

**Figure A2:**
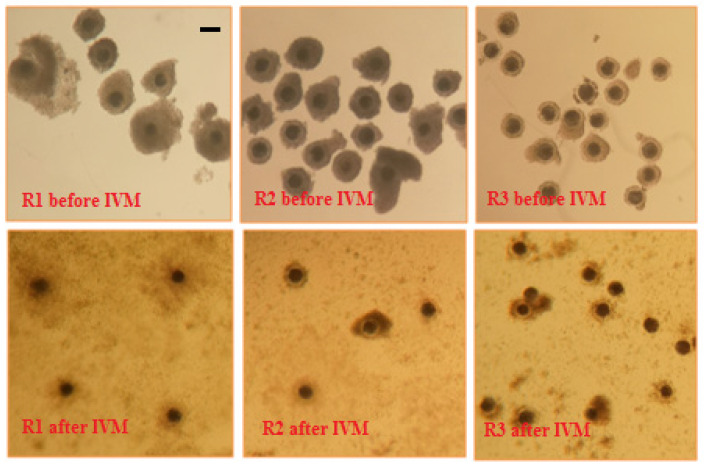
Aspects of porcine oocytes from rosmarinic acid group (R) classified according to their morphological aspects (class 1, 2 or 3) before and after IVM. Scale bar represents 100 µM. The scale bar is applicable to all panels (3.2×).

**Figure A3:**
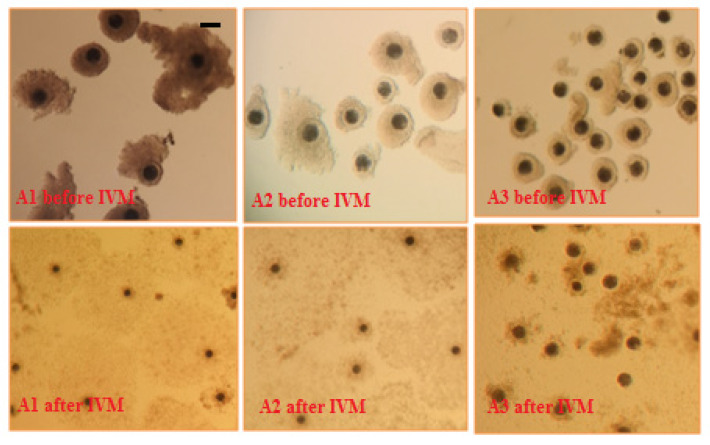
Aspects of porcine oocytes from ascorbic acid group (A) classified according to their morphological aspects before and after IVM. Scale bar represents 100 µM. The scale bar is applicable to all panels (3.2×).

When comparing the experimental groups with the control group, relative to the number of mature COC’s, an increase in their maturation sign is observed, with 7.83% (A1), 70.59% (A2) and 6.04% (A3) and respectively with 7.96% (R1), 42.75% (R2) and 24.63% (R3). By comparing the effects of the two antioxidants used according to the morphological class of COCs, we noticed that the use of ascorbic acid resulted in a better maturation of COC from class 2 by 27.84% than group R, and in class 3 rosmarinic acid had a better influence with 18.59% than group A. The main morphological aspects before and after the in vitro maturation of the sow COC in all groups can be seen in Figure A1, Figure A2 and Figure A3.

**Figure 1:**
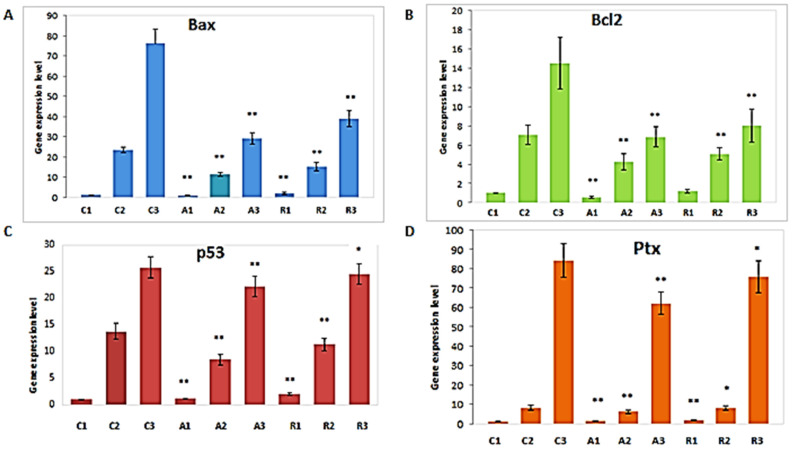
Bax (**A**), Bcl2 (**B**), p53 (**C**), and Ptx (**D**) gene expression levels in COC. Data represent the mean ± SD of the experiments, where * indicates a significant difference (*p* < 0.05) and ** a high significant difference (*p* < 0.01) of the experimental groups versus the control group, corresponding to their morphological class.

Generally, the qPCR expression averages of the genes Bax (A), Bcl2 (B), p53 (C), and Ptx (D) were significantly different (Kruskal–Wallis test statistics had a value 21.13, at *p* < 0.000) (Figure 1), where the significance levels of the *p* values are: *p* > 0.05, not significant; *p* ≤ 0.05, significant; *p* ≤ 0.01, very significant and; *p* ≤ 0.001, highly significant.

The results of the present study highlight the following aspects of Bax gene expression: when COCs were classified into morphological class 1, depending on the growth and maturation medium used, we can observe that the addition of vitamin C significantly reduces the expression of the Bax gene compared with the control group (A1 group vs. C1 group, *p* < 0.002). Similarly, the addition of rosmarinic acid in the R1 group significantly decreased the expression of Bax (R1 group vs. C1 group, *p* < 0.002). However, analyzing the influence of ascorbic acid and rosmarinic acid supplementation in the culture medium in the rest of the groups (A2, A3, R2, R3 groups, all at *p* < 0.004), we can see that they have a suppressive effect on Bax gene expression, compared with the control group (C2 and C3).

Evaluation of the expression of the anti-apoptotic gene Bcl2 reveals its significant decrease in the case of group A1 compared with group C1, *p* < 0.002, with no significant difference between group R1 and group C1 (*p* < 0.304). The same trend was observed for experimental class II compared with the control groups (A2 vs. C2 at *p* < 0.006 and R2 vs. C2 at *p* < 0.016) and III COC’s (A3 vs. C2 at *p* < 0.004 and R3 vs. C3 at *p* < 0.006).

**Figure 2:**
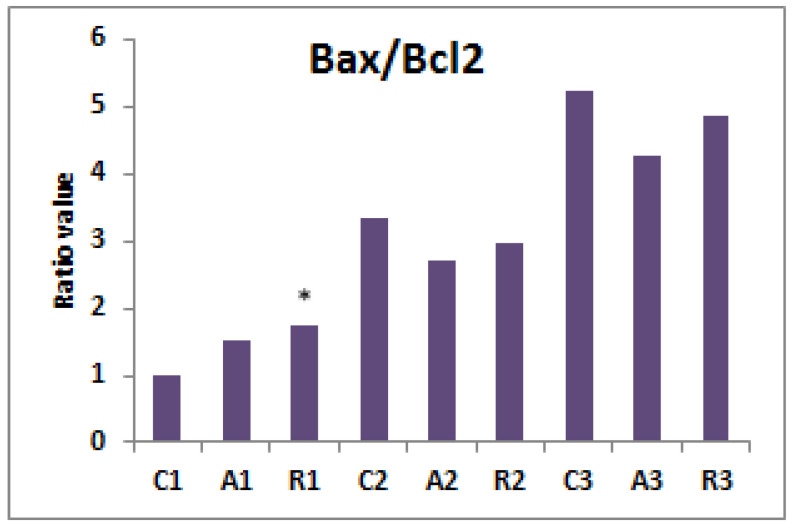
Bax/Bcl2 ratio value for each experimental group. *, *p* < 0.05.

In the case of all class I COC’s, regardless of the experimental group, a report of the expression of the Bax/Bcl2 genes was reported close to the value 1 (Figure 2), indicating minimum cellular stress and COCs being in a good condition, a fact evidenced by morphological studies, and so suitable for use in IVF. However, this aspect changes in the case of quality class 2, where the ratio increases in the case of groups cultivated in medium supplemented with ascorbic acid to a value of 2.67, and a value of 2.98 in the case of the media supplemented with rosmarinic acid, which confirms the presence of oxidative stress at the cellular level, which leads to a decrease in COC’s quality; however, despite this, the IVF process can still be attempted with these cells. In the case of COCs grown on non-supplemented medium, a value of this ratio of 3.32 is highlighted. This ratio is net in favor of Bax, with its expression counterbalanced precariously by the expression Bcl2, and so establishing cell apoptosis processes; therefore, these COCs do not have the necessary quality to be used for IVF. In the case of COC’s class 3, this report is clearly in favor of Bax expression, in the case of each experimental group studied, the apoptosis processes are initiated, and these cells are not viable for use.

Supplementation of the culture medium with ascorbic acid and rosmarinic acid in the case of COC class 1 leads to an increase in p53 gene expression compared with control group C1 (A1 vs. C1 at *p* < 0.002 and R1 vs. C1 at *p* < 0.002), but without determining significant morphological differences. In the COC class 2, there is a significantly lower expression of p53 in the case of groups supplemented with vitamin C and rosmarinic acid, compared with the cells in the control group that belong to the same quality classes (A2 vs. C2 at *p* < 0.004 and R2 vs. C2 at *p* < 0.016). Regarding class 3, there is a statistical decrease only between group A3 and C3 (A3 vs. C3 at *p* < 0.016). Compared with class I, high levels of p53 are observed in groups with class 2 (A2, R3) and 3 COCs (A3, R3), indicating a level of stress in these cells, but within the groups there are low levels of p53 (A1, R1).

In the case of supplementation, the usual environment with two compounds, there is a decrease in the expression of the Ptx 3 gene in the cells classified in groups A1 and R1 compared with groups C1 (A1 vs. C1, *p* < 0.002; R1 vs. C1 at *p* < 0.002), regarding class 2 of COCs the expression of the Ptx gene decreases significantly in group A2 (A2 vs. C2 at *p* < 0.016), but not in group R2 (R2 vs. C2 at *p* < 0.552). In class 3 the decrease was significant in group A3 versus C3 at *p* < 0.004, but not in group R3, where R3 versus C3 was at *p* < 0.109.

## 4. Discussion

In vitro embryo production is necessary in order to obtain high quality embryos that are used in reproductive biotechnology, as well as in research [4,10,12].

Because in assisted reproduction techniques, oocytes and embryos achieve their first stages of development in a non-natural environment, their defense system is affected, so these cells are no longer protected from oxidative stress. Under in vitro conditions, fluids in the oviduct, as well as the follicular fluid, play this defense role. Some of the adverse effects of oxidative stress on reproductive functions are mitochondrial dysfunction, DNA, RNA and protein damage, inhibition of ovulation-sperm fusion, and the inducing of blastomer apoptosis, especially in the blastocyst stage [1,2,11].

Therefore, to counteract these adverse effects, specialty studies indicate the need to supplement the culture media with certain concentrations of antioxidants [20,35,42,43].

The choice of antioxidants used in our experiments was based on encouraging literature data on their beneficial effects against oxidative stress during the maturation of oocyte in vitro and on cryopreservation of seminal material, with very little data in the literature on eth effects of rosmarinic acid on oocytes cultivated in vitro [30]. Thus, Luno et al. (2014) observed that, after defrosting, the boar sperm samples supplemented with rosmarinic acid (105 µM) had the lowest rate of DNA oxidation [32]. Regarding the use of rosmarinic acid in porcine oocyte maturation, Zhang et al. [30] observed an improvement in developmental competence when the rosmarinic acid was used in 5 µM concentration. The increase in quality of developmental competence was based on blastocyst formation rate, blastocyst hatching rate, blastocysts diameter, the total number of cells in blastocysts and somatic cell nuclear transfer (SCNT) embryos rate, and not on the quantification of genes expression.

Similar results have also been obtained with different antioxidants in different concentrations by Sadeesh et al., whose studies, both on oocytes and sperm from cattle [43], sheep [28], pigs [11,26,29,36] and carnivores [17], indicate that supplementation of maturation medium or cryoprotective medium, in the case of frozen semen, with antioxidants produces an improvement in the results.

L-ascorbic acid is used with good results in animal artificial reproduction techniques (ART) in both male [13,14,44] and female gametes [8,10,11,12]. Yu et al., 2018 concluded that 250 µM ascorbic acid can reprogram the methylation status of not only the DNA and histones, but also of the RNA, based on the methylation erasure in mature oocytes on 5mC and m6A nucleic acids and histones (H3K27me3) [16].

Analyzing the expression of the pro-apoptotic gene Bax we observed that the addition of ascorbic acid in the culture medium has a suppressive effect on Bax expression, favoring the protection of COC, in all the quality classes studied. Supplementing the culture medium with rosmarinic acid (group R2 and group R3), also bring a significant decrease in the expression of Bax gene compared with the control groups (groups C2 and C3), therefore these classes of COC’s could be used for IVF, based only on this gene quantification. This indicates that supplementation of growth medium with ascorbic acid and rosmarinic acid brings real benefits, including the reduction of cell stress.

An exception was identified in class 1, R1 and A1 groups where Bax gene expression was higher than in control group (C1 group). This could be possible because supplementation with the additives in the culture medium itself can cause stress on the COC, but, in conjunction with the Bax/Bcl2 ratio, it indicates that the stress produced has been compensated and that the COCs have adapted and thus are suitable for the IVF procedure.

The anti-apoptotic pattern of the Bcl2 gene is similar to that of Bax, indicating that supplementation of growth medium with ascorbic acid and rosmarinic acid brings real benefits, including the reduction of cell stress. Based on the literature data we know that the relationship between Bcl2/Bax appears to be an important aspect in the apoptotic process of granulosa cells [45]. The evaluation of the Bax/Bcl2 ratio represents a faithful index of the installation of oxidative stress at the cellular level, as well as the activation induction of the apoptotic process by activating the caspase pathway.

A Bax/Bcl2 ratio close to the value 1 in all class 1 COC indicates a minimum cellular stress and COC being in a good state. But this aspect changes in the case of quality class 2, where the ratio increases in the case of groups cultivated in medium supplemented with vitamin C to a value of 2.69 and a value of 2.96 in the case of the medium supplemented with rosmarinic acid. This confirms the presence of oxidative stress at the cellular level. Oxidative stress will produce a decrease of the COC’s quality, but with these cells, the IVF process can still be attempted.

In the case of the C2 and C3 groups grown in non-supplemented culture medium, a ratio value of 3.32 and 5.54 is highlighted, this ratio is net in favor of Bax, with its expression counterbalanced precariously by the expression Bcl2, establishing the cell apoptosis processes; therefore, these COC’s do not have the necessary quality to be used for IVF.

When analyzing the correlation between the morphological class 3 in all groups (C3, A3 and R3), this report is clearly in favor of Bax expression, with values of 5.24 (C3 group), 4.25 (A3 group) and 4.85 (R3 group). In these cases the apoptosis processes have been initiated and those cells are not viable for use.

The changes observed in the expression of the Bax and Bcl2 genes are in direct correlation with the observations made on COC at the morphological level.

Jang et al. (2004) observed in pig species that both antioxidant enzymes and apoptosis genes play an important role in the cultivation techniques of in vitro embryos [46].

The smallest values of Bax/Bcl2 ratio for groups A1 and R1 suggest the importance of the quality of the oocyte to be fertilized without the implication of supplementing the environment with antioxidants [47,48,49,50,51,52,53].

Similar results were obtained by Park et al. (2014) in cattle, thus the levels of apoptosis were higher in blastocysts obtained from oocytes matured in the environment without bovine follicular fluid supplementation (antioxidant capacity) (*p* < 0.05), with an influence on the subsequent embryo development [54].

The regulatory action of p53 may be direct, or it may act by interacting with other cellular proteins. When various stressor factors act on the cell, the p53 protein induces the overexpression of the pro-apoptotic Bax gene and inhibits the anti-apoptotic Bcl2 gene; all these processes together induce alterations in the mitochondrial membrane, thus decreasing cell viability.

The increase in p53 gene expression in the A1 and R1 groups compared with the C1 group has a possible explanation in the adaptation of cells to new factors introduced into the culture medium, and therefore inducing the activation of DNA repair mechanisms. In the A2 and R2 groups the expression of p53 gene was significantly lower than in the C2 group probably because those stressors acted on the cell and produced alterations at the cellular DNA level, activating the response of the cell defense and repair mechanisms that aim to maintain cell integrity and functionality; thus, the cells grown in supplemented medium, belonging to class 2, could be used in IVF with a satisfactory yield. In general activation of p53 is a response to various cellular stresses and induces cell cycle arrest, DNA repair and cell apoptosis. In groups A1, A2, A3, R1, but also in R2 and R3, only a significant decrease of the expression of the Ptx 3 gene compared with the control groups (C1, C2 and C3), is observed, meaning that the supplemented media is a proper environment for the cells. These are different to the results we see inside the groups, because in all class 3 (C3, A3, R3) the Ptx3 level is significantly increased (D in Figure 1) which represents an attempt of the cells to survive, the expression of this protein in the present case is trying to maintain the integrity of the cell matrix and implicitly the viability of COC, along with the overexpression of p53 with a repair role; however these mechanisms are overwhelmed and the cells from class 3 quality become unviable, a fact also highlighted by the morphological observations. Another important aspect of this study is that in class 3 COC, that included a large number of oocytes (214 COC, with 71.33/sample used for RNA extraction), but which obviously had a smaller number of cumulus cells, their genes expression indicates a higher cellular stress and important apoptosis. However, in morphological classes 1 and 2 there was a significant reduction in gene expression level, which can indicate that detected mRNA transcription level was mainly from oocytes or that BAX, BCL2, p53, Ptx3 mRNA transcription of that oocytes and cumulus cells have a similar level of expression, if we considered our previous research data [20].

Through DNA microarray analyses Hong et al. (2009) identified 34 regulated genes by p-53 in mouse and human fibroblasts and they observed that, if the p53 gene is knocked down, the induction of pluripotent stem cells is higher, indicating that p53 acts as a barrier to nuclear reprogramming, not only as a safeguard in tumorigenicity [55].Therefore, down-regulation of p53 in class 2 experimental groups (A2 and R2) indicates that antioxidants promote the survival of medium quality oocytes. Similar results were obtained when vimentin, an oocyte protein, was inhibited during porcine nucleolar reprogramming, the p53 pathway was activated, which means that this protein acts a genomic protector [56]. Heat stress is another factor that can influence oocytes quality, with up-regulation of p53 among target genes [57].

The abundance of genes related to cumulus expansion is another modality for evaluating oocyte maturation. Ptx3 has tended to be considered as a biomarker of oocyte quality [58], because it is involved in the assembly of the hyaluronan-rich matrix that forms around the oocytes, providing structural integrity to the cumulus matrix [59], but also plays a key role in the fertilization process [60]. Similar results have also been obtained in bovine oocytes with catechin polyphenols, epigallocatechin-3-gallate from green tea [61] or melatonin, through its antioxidant effects on bovine oocyte [62] or on pig oocytes [63].

Supplementation of the culture medium with antioxidants determined the increase in the number of matured oocytes from the point of view of the morphological assessment. Both the addition of ascorbic acid and rosmarinic acid in the culture medium produced a positive effect compared with the control groups, which is particularly noticeable in class I and II oocytes.

Evaluation of ascorbic acid (500 µM) and rosmarinic acid (105 µM), added in higher concentrations in the composition of in vitro medium maturation based on gene expression evaluation, suggests that these antioxidants can improve the developmental competence of pig oocytes.

## 5. Conclusions

Our results reveal a direct proportional relationship between the increase in the expression values of the studied apoptotic gene and the decrease in the quality of the oocytes capable of being used in the in vitro fertilization process.

The level of expression of the Bax gene, the pro-apoptotic gene, tends to increase in all COCs inversely proportional to the quality of oocytes, so that level 3 is the highest, indicating that the cellular adaptation process in this class has been exceeded. Regarding the Bcl-2 gene, significantly decreased expression levels (*p* < 0.002) can be observed in class I oocytes supplemented with antioxidants. This means that both the added antioxidants and the quality of the oocyte play an important role in maintaining cell viability.

Compared with the control groups, both antioxidants used in this study determined a better expansion of cumulus cells in both species, especially in class 1 and class 2 COCs. The level of maintenance of cell homeostasis, reflected in the ratio of Bax/Bcl-2, which has a supraunitary value, indicates the apoptotic processes for all class 3 oocytes.

Further research is also required on in vitro porcine embryos to validate the beneficial effects of ascorbic acid (0.5 mM) and rosmarinic acid (105 µM) as antioxidants and, therefore, to contribute to improving IVF production of pig embryos, something which would represent an important step in obtaining transgenic pigs.

## Data Availability

Not available.

## Sample Availability

Samples of the compounds are not available from the authors.
